# Spanish Adaptation and Psychometric Properties of the Sport Motivation Scale-II with High School Physical Education Students

**DOI:** 10.3390/ijerph15122768

**Published:** 2018-12-06

**Authors:** Antonio Granero-Gallegos, Manuel Gómez-López, Juan González-Hernández, Antonio Baena-Extremera, María del Mar Ortiz-Camacho

**Affiliations:** 1 Department of Education, Faculty of Education Sciences, University of Almeria, 04120 Almería, Spain; agranero@ual.es; 2 Department of Physical Activity and Sport, Faculty of Sport Sciences, University of Murcia, 30720 Santiago de la Ribera, Murcia, Spain; 3 Department of Personality, Evaluation and Psychological Treatment, University of Granada, 18071 Granada, Spain; jgonzalez@ugr.es; 4 Department of Didactic of Corporal Expression, Faculty of Education Sciences, University of Granada, 18071 Granada, Spain; abaenaextrem@ugr.es (A.B.-E.); mmortiz@ugr.es (M.d.M.O.-C.)

**Keywords:** physical education, motivation, psychometric, self-determination, SMS-II

## Abstract

The objective of this work was to provide evidence on the dimensionality of the Spanish version of Sport Motivation Scale II adapted to physical education (SMS-II-PE) in one of its hypothesized models (6 factors). A sample of 1055 secondary education students of the Region of Murcia, Spain, aged between 12 and 17 years, was used. As an instrument, the SMS-II-PE was used. The factorial structure was examined with confirmatory factor analysis, and internal consistency and invariance by sex were evaluated. The results confirmed that the Spanish model of 6 factors and 18 items presented good adjustment indicators and is valid for its application in the educational field of Physical Education lessons.

## 1. Introduction

Self-determination theory (SDT) is an approach to human motivation which highlights the importance of the evolution of internal human resources for personality development and behavior self-regulation [1].

Motivation is understood as one of the variables that has the most influence on the educational context [2,3,4]. In addition, measuring this construct with current instruments is of great interest, and SDT is one of the most used theories in research, both from the sporting and educational field [5,6]. Motivation in its relationship with self-determination is understood as a continuum in which the behavior of the subject is ordered from the highest to the lowest degree of self-determination. This theory establishes the existence of three manifestations of intrinsic motivation: the intrinsic motivation for knowledge, for achievement, and for stimulating experiences [7]. On the opposite side, extrinsic motivation, which refers to carrying out behaviors for instrumental reasons or external sources, stands. In it, according to Deci and Ryan [5,8], it can be distinguished from a greater to lesser degree of self-determination: integrated regulation, identified regulation, introjected regulation, and external regulation. Finally, there is the amotivation or relative absence of motivation, in which the subject is not motivated, neither intrinsic nor extrinsically [9].

In recent years, the research in relation to the importance of intrinsic motivation in physical education students is of great relevance (e.g., [3,10]) especially since this implies greater interest and participation in the sessions, which leads to an increase in physical activity in their free time [11,12], with all the benefits that this entails. Literature has shown a positive relationship between the practice of physical activity and improving the academic performance of children and adolescents, and even suggests that there are other benefits, such as those related to health, compared to children who do not practice sport in their free time [10,13,14,15,16,17,18,19,20,21,22].

Numerous research studies have focused on discovering students’ motivational processes and levels, such as motivational climate [23], due, among other reasons, to the decisive educational connotations associated with high motivation levels: academic satisfaction [10,24], improvement in grades [25], and even improvement in foreign language learning [26], among others.

A series of different instruments have been designed and used over the last few decades, in order to determine the degree or level of motivation in students. Many of them are based on a French scale, *l´Échelle de Motivation en Éducation* (EME) by Vallerand et al. [7], later validated in English as the Academic Motivation Scale (AMS) [27]. This first version included 28 items grouped in seven factors, and studied motivation in the educational context. Then, in 1995, and with the AMS as a reference point, Brière et al. [28] validated the *Échelle de Motivation dans les Sports* (ÉMS), an instrument, in this case, designed to measure self-determination in sports. This instrument included the three representations of intrinsic motivation (IM) (knowledge, achievement, and stimulation) and three of the four types of extrinsic motivation (EM) (identified regulation or IDR, introjected regulation or INTR, and external regulation or EXTR), thus excluding integrated regulation (INTEGR) as suggested by Brière et al. [28] and Pelletier et al. [29] and, finally, amotivation (AMO).

In the light of this study, Pelletier et al. [29] created the definitive version in English, the Sport Motivation Scale (SMS), which included 28 items and seven dimensions, each with four items. The SMS has been widely used and studied over the last few years, and there is some controversy around its dimensionality and reliability. Thus, some studies have supported and reinforced the seven-factor structure with the results obtained in their factor analysis and carrying out validation in various countries: Doganis [30] in Greece, Núñez, Martín-Albo, Navarro and González [31] in Spain, Burtscher, Furtner, Sachse and Burtscher [32] in Germany, Bara et al. [33] in Brazil, and Paic, Kajos, Meszler, and Prisztóka [34], in Hungary, among others. Other authors studied the possibility of estimating models with three, five, and six SMS factors. Specifically, Alexandris, Tsorbatzoudis and Grouios [35], Martens and Webber [36], Zahariadis, Tsorbatzoudis and Grouios [37] and Guzmán, Carratalá, García-Ferriol and Carratalá [38], the latter, in Spain, concluded that the results obtained by analyzing IM, EM, and AMO each in isolation revealed better fit indices than for the whole model. On the other hand, Li and Harmer [39] and Ntoumanis [40] concluded that it was possible to use a five-dimension model combining the three representations of IM in one dimension. Finally, Mallett et al. [41] showed that from a statistical and theoretical point of view, it is possible to estimate a six-factor SMS version. These studies reveal that the different analyses present some factor validity problems (e.g., [37]) and low internal consistency (e.g., [29,42]).

In addition to the above, one of the main current lines of work on the SMS is the inclusion of the INTEGR factor which, as mentioned before, was not included in the French or English version of the SMS. Specifically, the INTEGR is the most complex factor in terms of its explanation in achievement motivation theory, as it implies not only identification with the importance of behaviors (IDR), but also integrating these identifications with other aspects of ourselves, such as values, goals, personal needs and identity in a harmonious and consistent way (e.g., [41,43]). In this regard, Mallett, Kawabata, and Newcombe [44] suggested that the scale should be revised in order to include an INTEGR measurement as, without it the scale, it did not represent all the constructs of the SDT framework, plus some items had certain problems. In light of these suggestions, other versions to measure motivation with an INTEGR factor appeared, such as the Behavioural Regulation in Sport Questionnaire (BRSQ, BRSQ-8 and BRSQ-6) by Lonsdale, Hodge, and Rose [45]. However, it also presented consistency problems. Finally, Pelletier et al. [46] revised the SMS and created the SMS-II (18 items in six factors); they included INTEGR and eliminated some items, leaving, for example, IM in one only factor (IM, INTEGR, IDR, INTR, EXTR, and AMO). Thus, they solved some problems connected with these particularities and obtained good values in terms of internal reliability and validity of the construct. 

The psychometric properties of this new version were object of analysis for adaptations to other languages and cultures, but in sports contexts in all cases: Swedish [47]; the Brazilian-Portuguese context [48]; Chinese [49]; French [50]; Turkish [51]; and also Spanish versions in Mexico [52] and Spain [53]. 

In the case of adaptation to physical education (PE) in schools, the first version of the SMS has been used in Spain so far. Some researchers (e.g., [54]) used the seven-factor version by Núñez et al. [31] in high school students, and obtained good consistency, reliability, and fit indices. Along these lines, Granero-Gallegos et al. [55] assessed the internal consistency and convergent validity of the three possible models of the SMS (three, five, and seven factors, INTEGR excluded) in the Spanish context, and found good fit indices in all of them in physical education students. However, they recommended the seven-factor model, as it obtained the best values for this subject. Despite these advances, there is no current validation of the SMS-II for physical education and, therefore, its adaptation to the educational context presented here is an interesting contribution to the international literature in this field.

In view of the above, the use of the most updated version of this instrument is important to measure motivation among high school students, and the question is whether the Spanish version of SMS-II will be a valid and reliable instrument for that. Thus, this research had two objectives: (a) to contribute evidence regarding the dimensionality of the Spanish version of the SMS-II adapted to physical education with a sample of high school students in Spain. To this aim, we carried out a study of each of the items in the scale and a confirmatory factor analysis, internal consistency being also analyzed, as well as temporary stability and gender invariance; (b) to prove criterion validity by means of gender difference analysis in all the dimensions of the scale. The hypothesis of this research is that the SMS-II adapted to physical education has adequate indices of fit of validity and internal consistency, and will be invariant by gender.

## 2. Method

### 2.1. Participants

A non-probabilistic convenience sampling was used on this research [56,57]. A total of 1055 high school students (519 boys; 536 girls) within a 12–17 year age range (boys: *M* = 13.87; *SD* = 1.42; women: *M* = 13.93; *SD* = 1.37) from seven educational centers in the Region of Murcia participated in this study. In order to analyze the temporal stability of the scale, we used an independent sample of 63 high school students (31 boys; 32 girls) within a 12–17 year age range (*M* = 13.64, *SD* = 1.23).

### 2.2. Measurement Instruments

Sport Motivation Scale-II adapted to physical education (SMS-II-PE). A Spanish adaptation (see Annex) of the *Sport Motivation Scale-II* by Pelletier et al. [46] was used. The instrument includes a total of 18 items to measure individual level of motivation towards physical education distributed in six dimensions (three items per dimension): *intrinsic motivation* (e.g., “Por el placer que siento mientras realizo actividad físico-deportiva”), *integrated regulation* (e.g., “Porque la práctica de actividad físico-deportiva es parte fundamental de mi vida”), *identified regulation* (e.g., “Porque la actividad físico-deportiva es una forma para desarrollarme”), *introjected regulation* (e.g., “Porque me sentiría mal si no participara y me esforzara en las clases”), *external regulation* (e.g., “Porque obtengo recompensa de la gente que me rodea cuando lo hago”) and *amotivation* (e.g., “Antes participaba y me esforzaba en las clases, pero ahora me pregunto si debo continuar haciéndolo”). The scale used was preceded by the following introductory sentence: “Participo y me esfuerzo en las clases de Educación Física…” The answers were collected on a Likert-type scale from 1 (*I strongly disagree*) to 7 (*I completely agree*).

### 2.3. Procedure

First, the items of the different subscales of the *Sport Motivation Scale-II* [46] were translated using back-translation, as suggested by authors like Muñiz, Elosua, and Humbleton [58]. Two translators carried out the translation of the original SMS-II into Spanish. Following that, two different translators translated the items back to their original language (back-translation). The quality of the translation was assessed, bearing in mind the agreement with the original version. The final version obtained was analyzed by a group of four experts [59] in physical education and activity, so as to guarantee the adequate design of the items in terms of the dimensions that the construct intended to measure, and to maintain the original meaning [58]. 

The experts were given a specification table of the items [60] which collected the semantic definition of the construct to be evaluated, and that of its component. They were then shown a list of items once the original had been adapted, for them to make a judgment in terms of suitability and comprehensibility on a scale from 1 (*I strongly disagree*) to 4 (*I completely agree*). Furthermore, there was a section for them to make notes and general observations about each of the items, and they had the possibility of providing an alternative wording of each item if they thought it appropriate. The items reaching a <2.5 median score, both in suitability and comprehensibility, were revised. When an item was not judged by at least three of the four experts as within the theoretical dimensions of the scale, it was revised, once more, to analyze potential problems before proposing an alternative wording that summarized the theoretical dimension in a more clear and accurate way. Global agreement of the four experts regarding the suitability and comprehensibility of the items was measured through the intraclass correlation coefficient (ICC), based on a mixed-effects model and assuming an absolute agreement definition. The values obtained were ICC = 0.78 in suitability and ICC = 0.83 in comprehensibility.

Furthermore, the interquartile range was used as the criterion to measure dispersion in the four experts’ agreement. If the difference of the percentile 3 in relation to percentile 1 was equal to 0 or 1, the item was accepted and/or slightly modified; if this difference was between 1 and 2, the item was revised and reformulated, while if the difference was larger than 2, dispersion amongst the experts was deemed too high, and the item was rejected. The experts’ comments on the instructions and wording resulted in minor changes. The new version was administered to 83 high school students aged 12–16 years old. The students stated their complete understanding of the items and, after a final revision by the research team, the final Spanish version of the SMS-II adapted to PE (SMS-II-PE) was completed. 

Following that, the administrators of different high schools were contacted to request their collaboration, and the PE teachers who decided to participate on the research purpose were briefed on the procedure. Given that the students were minors, consent was obtained from their parents/guardians for participation in the study. The students were informed of the purpose of this research, and of their rights as participants, based on the Declaration of Helsinki [61]. The instrument was administered in class, in the presence of the main researcher. The importance of the study and the way to complete the scale was explained, and doubts regarding the meaning of the items were solved during the administration process. The questionnaires were individually collected as participants finished, so as to detect errors and verify that all items had been answered. Approval was obtained from the Bioethics Committee of the Universidad de Murcia for this research work.

### 2.4. Data Analysis 

First, a calculation was made of the descriptive and homogeneity statistics of the items and of the internal consistency of each of the factors in the scale. Then, based on the structure of the original instrument and with the aim of checking its current structure, we carried out a confirmatory factor analysis (CFA). Given that Mardia’s coefficient was high (70.76), the method of maximum verisimilitude was used in the CFA, together with bootstrapping. The regression models were evaluated using a combination of fit indices: chi-square (χ^2^)/degrees of freedom (df) (χ^2^/gl) ratio, as χ^2^ is highly sensitive to sample size [62]; the confirmatory fit index (CFI); Tucker–Lewis index (TLI); root mean square error of approximation (RMSEA) plus its confidence interval at 90%, and standardized root mean square (SRMR). In the case of χ^2^/df, <5.0 values are considered good fit values, and <2.0 ratios are considered excellent fit values [63]. In terms of CFI and TLI, values ≥0.90 are considered good fit values [64]. Error indices are considered acceptable with values equal to or lower than 0.07 for RMSEA, and 0.08 for SRMR [64]. The modification indices and the standardized residuals matrix obtained in the CFA were analyzed in order to detect potentially problematic items. Following Byrne [65], items with high values in standardized residuals (>±2.58) were considered for potential elimination.

In terms of internal consistency, reliability was calculated with McDonald’s ω and Cronbach’s alpha. Thus, we covered the potential limitations of Cronbach’s alpha [66], especially when the variables consist of a small number of items [67], as is the case of the instrument analyzed in this study. McDonald’s ω—as opposed to the alpha coefficient—takes into account the factorial loads, which makes calculations more stable, and reflects the actual reliability level regardless of the number of items in the variable (see, [66]). Internal consistency values (ω) are deemed acceptable within the 0.70 and 0.90 range [68], though Katz [69] accepts >0.65 values. Further to this, temporal stability was analyzed with the intraclass correlation coefficient (ICC) and its IC at 95%, with ≥0.70 values being accepted as adequate [70].

The heterotrait–monotrait ratio of correlations (HTMT) was obtained in order to support the discriminant validity of the construct, with values <0.85 [71] being accepted as adequate, though some authors accept <0.90 values [72].

Then, a gender invariance factor analysis was carried out. Four progressively restricted multi-group models were specified [73]: (a) Model 1 (structural); (b) Model 2 (metrics); Model 3 (scale); and Model 4 (strict). Following Cheung and Rensvold [74], we took into account that the invariance null hypothesis should be rejected with >0.01 falls in CFI values (ΔCFI contrast test) between successive restricted models. Finally, in terms of criterion validity, gender differences across the different SMS-II dimensions were measured through a variance univariate analysis using SPSS v.22 and AMOS v.22.

## 3. Results

### 3.1. Items Analysis 

The item factor distribution in the original Pelletier et al. [46] instrument was maintained in the statistics items’ analysis. Furthermore, the procedure described in Carretero-Dios and Pérez [75] was used for the analysis of each of the items evaluated. We also analyzed whether the internal consistency of the scale increased with the elimination of any of the items in each dimension, as well as the requisites established by Nunnally and Bernstein [76], corrected total-item correlation coefficient (CTICC-c) ≥ 0.30, standard deviation (*SD*) > 1, and all the answer options used at any given point. It is also worth noting that the asymmetry and kurtosis indices are within the −0.94 and 1.23 range (Table 1). Furthermore, following Taylor, Ntoumanis, and Standage [77], we considered that when a factor is composed of a small number of items (in this case, each one is composed of three items) a <0.70 internal consistency index can be accepted as adequate. 

### 3.2. Confirmatory Factor Analysis

We then carried out a CFA, bearing in mind the original structure of the scale. The original model with 18 items and six correlated factors (SMS-II-PE) showed good fit indices: χ^2^ = 481.57, *p* < 0.001; χ^2^/*df* = 4.01; CFI = 0.94; TLI = 0.95; RMSEA = 0.054 (IC90% = 0.049, 0.059), RMSR = 0.047. All the parameters were positive and significant (*p* < 0.05), and virtually all items had >0.60 factorial loads, with the exception of four items: 3, 1, 7, and 2 (see Table 2), though they reached >0.50.

### 3.3. Reliability Analysis

The instrument’s reliability was assessed through an internal consistency analysis and a temporal stability analysis. Cronbach’s alpha and McDonald’s ω reached adequate reliability indices (Table 2). In the case of the temporal stability analysis, the instrument was applied twice to an independent sample, with a four-week interval between data collections. The intraclass correlation coefficient (ICC) was obtained to estimate temporal stability for the different subscales, and the values were over 0.84.

As to discriminant validity, the HTMT ratio correlation value between the different dimensions were <0.85 [71], except for IM with INTEGR and with IDR, which reached 0.92 and 0.94, respectively. These high correlation values can also be observed in Table 2, which shows the correlations between the different latent variables and still show excessively high correlation values (>0.85; see [78]). 

### 3.4. Invariance Analysis

A gender invariance analysis was carried out (Table 3) in order to simultaneously test the equivalence of the factorial structure in both subgroups. No statistically significant differences were found between Model 1 (model without restrictions) and Model 2 (invariance in measurement weights) (*p* = 0.518), or between Model 1 and Model 3 (invariant structural variances and covariances) (*p* = 0.293). However, statistically significant differences were observed between Model 1 and Model 4 (invariant measurement residuals) (*p* = 0.000). According to Byrne [65], a lack of statistically significant differences between Model 1 and 2 constitutes a minimum criterion to accept invariance in the model. Moreover, we took into account that the fall in the CFI values were <0.1 (ΔCFI contrast test) across the different models, which, following Cheung and Rensvold [74], means the model is proven to be gender-invariant.

### 3.5. Gender Differences Analysis 

Gender differences were assessed in the different dimensions of the SMS-II-PE using univariate variance analysis (see Table 4) for criterion validity. Statistically significant differences were found in all factors, except for INTR and AMO. Boys showed higher median values than girls in all the dimensions except AMO. However, the values of the partial squared statistic (effect-size) are low, even where they appear as statistically significant.

## 4. Discussion 

The objectives of this study were to present evidence of the dimensionality of the Spanish version of the SMS-II adapted to physical education, and to verify its internal consistency, temporal stability, discriminant and criterion validity, and gender invariance after adaptation into Spanish. 

As reflected in the Introduction section, there are previous versions of the SMS in Spanish, both in the sports context [79] and in physical education [55]. However, in terms of the new version—the SMS-II by Pelletier et al. [46]—only adaptations of this instrument to sports exist up to now in Spanish [52,53]. It is worth noting here that this SMS-II version involves a reduction of the original instruments to 18 items, with the INTEGR factor as a new inclusion, which had been criticized by Mallet et al. [41,44], among others. This new version would allow for a thorough evaluation of all the SDT constructs in sports (and now also in PE). Furthermore, recent studies have contributed, with new analyses of the SMS-II factorial structure aiming at revealing potential improvements in terms of the original SMS dimensionality and reliability, which had been the subject of criticism. Therefore, the adaptation to physical education, in this study, covers an important need for teachers and researchers in this field. 

In relation to the hypothesis, the results obtained after and analysis of the psychometric properties show adequate validity and reliability levels in the six first-order factors with 18 items, except for AMO and INTEGR, which is line with the results in Viciana et al. [53], Pineda-Espejel et al. [52], and Beddoes [80], in demotivation and introjected regulation). However, it is worth noting that in Viciana et al. [53], for sports, Cronbach’s alpha, Omega, and AVE values for INTEGR (0.686, 0.696, 0.434, respectively) and EXTR (0.536, 0.552, 0.300, respectively) were inadequate. By contrast, all the SMS-II-PE factors reached good reliability McDonald’s ω values, and even Cronbach’s alpha reliability values can be considered adequate, given the small number of items in each factor [77]. In terms of stability, the instrument shows adequate temporal stability. This point is also worth noting as a contribution of this study to the international scientific literature in the education field. 

The CFA results were also adequate, both for absolute and incremental fit indices, in line with other studies, such as Pelletier et al. [46], Pineda-Espejel et al. [52], and Viciana et al. [53]. The latter study found similar RMSEA and CFI values, though they improved in their five-factor model with the elimination of some problematic items (RMSEA = 0.044; CFI = 0.966). In view of these data, it would be highly interesting to carry out future research work following the proposal in Viciana et al. [53], who presented the possibility of reformulating items 1, 7, and 15 for PE (if the aim is to consolidate the six-factor structure in [81]), or even verify if one of five dimensions would have a better fit. 

In relation to factorial loads, we obtained values under 0.60 for some of the scale items (item 3 in IM, items 1 and 7 in INTR, an item 2 in AMO). These results are partially reflected in previous research, with different items reaching low factorial loads [46,48,53]. Some studies even carried out their analysis excluding certain items (item 16) [52], and obtained better CFA values. It is worth noting also that Stenling et al. [47] suggested the exclusion of items 7 and 8, whereas Li et al. [49] suggested deleting 1, 2, 15, and 18.

These previous results reveal that these items still need to be revised in order to improve scale validation. Moreover, discriminant validity showed some inadequate values, specifically those between IM, INTEGR, and IDR, in line with Nascimento et al. [48] in the Portuguese-Brazilian version, and with Viciana et al. [53]. Similarly, Li et al. [49] found problems in discriminant validity between IDR and INTEGR in one of their studies. This shows that this instrument should be revised, again, as there are high correlation values between factors of the same construct, which might reflect a comprehension problem.

Answering the hypothesis in relation to gender invariance, the data reveal that this instrument is invariant when it comes to physical education, as is the case with the SMS-II in Li et al. [49], Pelletier et al. [46], and Viciana et al. [53] in the sports context. These results therefore show robustness in terms of gender invariant behavior when this instrument is used for sample analysis.

Finally, in terms of criterion validity highlighted in the hypothesis, the data show higher IM in boys, results in line with those in previous research with SMS (e.g., [82]). Other papers have shown high values in boys in IM, IDR, INTR, EM, and AMO [83]. In addition, EM values were also contrasted, and in line with research on students of Ortiz-Camacho et al. [83], and on sports like the researches carried out by Núñez et al. [31], and Pelletier et al. [29], the data reveal significantly lower values in girls. Furthermore, the results reveal differences in all the factors, except for INTR and AMO. It should be taken into account that INTR is the most self-determined expression of extrinsic motivation and, according to the literature, it is a latent construct difficult to measure [84]. This is mainly due to the fact that it is complex for students to discern when a behavior has been internalized [85]. Previous studies on physical education (e.g., [55]) did find statistically significant differences in all the dimensions of the SMS, including INTR and AMO.

The differences by gender of each factor and the effect sizes are found in the RINTE and in the REXT. Regarding this, we can see, in Table 2, the high correlation between factor 1 and 2, as Pelletier et al. [46], who states that it is normal to find high correlations between subscales that are close to the continuum, and weaker correlations with the elements that are further apart. This means that there are greater effect sizes in the factors that are just attached to the other dimensions, since it is difficult for boys and girls to distinguish between one and the other.

## 5. Conclusions

To conclude, this paper shows that the hypothesis has been corroborated. It is safe to state that this study verifies the SMS-II-PE’s validity and reliability, in the same way as Pelletier et al. [46], among others, verified this for sports. Therefore, its use is recommended to measure physical education students’ motivation Spanish, as it is as valid as the original version (SMS), plus it is easier (shorter) to complete.

The interesting results in this study and its educational implications notwithstanding, some limitations should be taken into account. First, the sample is not probabilistic and, therefore, the results cannot be generalized to the student population. Second, some of the items generated problems, which could be solved with a new formulation and with the support of concurrent validity. To this aim, given that scale development is a continuous process, as noted by other authors [44,85], it would be advisable to carry out further studies to examine and make advances on the SMS-II-PE, as suggested also by Li et al. [49], by comparing it with a five-factor model or with other versions, such as the BRSQ.

## Figures and Tables

**Table 1:** Descriptive statistics, internal consistency and homogeneity (*n* = 1055).

Factors:	*M*	*SD*	CCIT-c	α without Item	Q1	Q2
*Intrinsic Motivation*						
Item-3. Porque es muy interesante aprender cómo puedo mejorar	5.56	1.45	0.45	0.60	−0.92	0.24
Item-9. Porque me permite descubrir nuevas actividades físico-deportivas	5.42	1.62	0.52	0.50	−0.94	0.07
Item-17. Por el placer que siento mientras realizo actividad físico-deportiva	5.22	1.77	0.46	0.59	−0.67	−0.49
*Integrated Regulation*						
Item-4. Porque la práctica de actividad físico-deportiva refleja la esencia de quien soy	4.29	1.82	0.56	0.61	−0.17	−0.91
Item-11. Porque la práctica de actividad físico-deportiva es parte fundamental de mi vida	4.93	1.80	0.55	0.61	−0.54	−0.80
Item-14. Porque la práctica actividad físico-deportiva hace que viva según mis principios más profundos	4.08	1.86	0.51	0.67	−0.03	−0.97
*Identified Regulation*						
Item-6. Porque creo que es una buena manera de desarrollar aspectos que valoro de mí mismo/a	4.94	1.69	0.58	0.70	−0.65	−0.37
Ítem-12. Porque la actividad físico-deportiva es una forma para desarrollarme	5.13	1.63	0.59	0.69	−0.71	−0.26
Item-18. Porque es una de las mejores formas de desarrollar otros aspectos de mí mismo/a	5.22	1.64	0.62	0.65	−0.74	−0.22
*Introjected Regulation*						
Item-1. Porque me sentiría mal conmigo mismo/a si no dedicara tiempo a la práctica de actividad físico-deportiva	4.51	1.87	0.46	0.56	−0.30	−0.92
Item-7. Porque me sentiría mal si no participara y me esforzara en las clases	4.63	1.95	0.50	0.49	−0.44	−0.99
Item-16. Porque me siento mejor conmigo mismo cuando participo y me esfuerzo en clase	5.59	1.64	0.43	0.60	−0.87	0.34
*Extrinsic Motivation*						
Item-5. Porque la gente que me importa se molestaría conmigo si no lo hiciera	2.45	1.81	0.56	0.53	1.12	0.12
Item-8. Porque creo que los demás desaprobarían que no lo hiciera	2.83	1.83	0.52	0.58	0.71	−0.63
Item-15. Porque obtengo recompensa de la gente que me rodea cuando lo hago	3.00	1.90	0.44	0.68	0.64	−0.74
*Amotivation*						
Item-2. Antes participaba y me esforzaba en las clases. pero ahora me pregunto si debo continuar haciéndolo	2.25	1.74	0.31	0.60	1.10	0.86
Item-10. Tengo la impresión de que no soy capaz de tener éxito en las actividades físico-deportivas que realizo en clase	2.79	1.95	0.42	0.46	0.82	−0.60
Item-13. Realmente no me siento capacitado/a para la práctica físico-deportiva	2.17	1.66	0.47	0.38	1.23	1.02

Note: *n* = sample; *M* = mean; *SD* = standard deviation; CCIT-c = corrected coefficient of item-total correlation; α = Cronbach’s alpha; Q1 = skewness; Q2 = kurtosis.

**Table 2:** Parameters of the model of six correlated factors (SMS-II-PE).

	IM	INTEGR	IDR	INTR	EM	AMO
Items	B (standardized)	B (standardized)	B (standardized)	B (standardized)	B (standardized)	B (standardized)
3	1.00(0.54) *					
9	1.26(0.61) *					
17	1.52(0.68) *					
4		1.00(0.68) *				
11		1.06(0.73) *				
14		0.94(0.63) *				
6			1.00(0.70) *			
12			0.96(0.70) *			
18			1.05 (0.76) *			
1				1.00(0.54) *		
7				1.06(0.56) *		
16				1.32(0.73) *		
5					1.00(0.72) *	
8					0.94(0.67) *	
15					0.87(0.60) *	
2						1.00(0.53) *
10						1.32(0.61) *
13						1.32(0.68) *
Correlations						
IM		0.90(0.87) *	0.90(0.90) *	0.75(0.79) *	0.22(0.35) *	−0.29(−0.47) *
INTEGR			0.85(0.82) *	0.85(0.70) *	0.76(0.54) *	−0.26(−0.33) *
IDR				0.92(0.70) *	0.46(0.39) *	−0.30(−0.23) *
INTR					0.33(0.42) *	−0.26(−0.25) *
EM						0.56(0.43) *
AMO						
Reliability						
*α*	0.68	0.72	0.76	0.78	0.72	0.64
ω	0.89	0.87	0.85	0.86	0.86	0.87

Note: * *p* < 0.01; *α* = Cronbach’s alpha; ω = McDonald’s omega; IM = intrinsic motivation; INTEGR = integrated regulation; IDR = identified regulation; INTR = introjected regulation; EM = extrinsic motivation; AMO = amotivation.

**Table 3:** Invariance by gender.

Models	χ^2^	df	χ^2^/df	Δχ^2^	Δdf	CFI	TLI	SRMR	RMSEA(IC90%)
Model-1	586.54	236	2.49	-	-	0.94	0.94	0.047	0.038(0.034–0.041)
Model-2	597.66	248	2.41	11.13	12	0.94	0.95	0.048	0.037(0.033–0.040)
Model-3	623.44	269	2.32	25.77	21	0.94	0.95	0.052	0.035(0.032–0.039)
Model-4	696.84	289	2.41	73.40	20	0.93	0.95	0.052	0.037(0.033–0.040)

Note: χ^2^ = chi-square; df = degrees of freedom; CFI = confirmatory fit index; TLI = Tucker–Lewis index; SRMR = standardized root mean square; RMSEA = root mean square error of approximation.

**Table 4:** Univariate analysis by gender.

Male (*n* = 519)			Female (*n* = 535)				
Factors	*M*	*SD*	*M*	*SD*	F	p	η^2^
IM	5.45	1.26	5.21	1.24	9.30	0.002	0.02
INTEGR	4.73	1.41	4.15	1.46	42.16	0.000	0.06
IDR	5.29	1.34	4.91	1.36	20.25	0.000	0.03
INTR	4.97	1.43	4.85	1.37	1.78	0.082	0.00
EM	3.12	1.51	2.41	1.31	65.46	0.000	0.08
AMO	2.38	1.35	2.43	1.30	0.309	0.578	0.00

Note: *M* = mean; *SD* = standard deviation; *n* = sample; IM = intrinsic motivation; INTEGR = integrated regulation; IDR = identified regulation; INTR = introjected regulation; EM = extrinsic motivation; AMO = amotivation; η^2^ = partial squared statistic
